# Investigation of occurrence patterns of respiratory syncytial virus A and B in infected-patients from Cheonan, Korea

**DOI:** 10.1186/s12931-020-01456-3

**Published:** 2020-07-18

**Authors:** Ga-Yeon Kim, Insoo Rheem, You Hyun Joung, Jae Kyung Kim

**Affiliations:** 1 Department of of Public Health, Dankook University Graduate School, Cheonan, South Korea; 2 Department of Laboratory Medicine, Dankook University College of Medicine, Cheonan, South Korea; 3 Department of Biomedical Laboratory Science, Dankook University College of Health Sciences, Cheonan, South Korea

**Keywords:** Respiratory syncytial virus, Respiratory virus, Epidemiology, Real-time RT-PCR

## Abstract

**Background:**

Respiratory infections caused by viruses affect the lower respiratory tract; these infections are severe in patients with underlying diseases and can even lead to death. Respiratory syncytial virus (RSV), one of the causative agents of respiratory viral infections, is the most common cause of pneumonia and bronchiolitis in children and adults.

**Methods:**

Respiratory specimens (nasopharyngeal aspirate, nasal swab, throat swab, etc.), which were sent to the Department of laboratory medicine from January 2012 to December 2018 for detection of respiratory viruses via real time reverse transcription PCR (Real time RT-PCR) were used in this study. RSV detected by real-time RT-PCR were analyzed on the basis of co-infection, sex and age of the patients, and year and month of sample collection.

**Results:**

During the study period, we observed that the RSV detection rate was 12.8% (*n* = 1150/9010); the detection rate of RSV-A (7.1%) was higher than that of RSV-B (5.8%). The detection rate of RSV was the highest at 36.5% in December, and RSV-A and RSV-B were in vogue every year. Co-infection rate of RSVs was the highest in the patients over 80 years of age; RSVs showed the highest Co-infection with Rhinoviruses.

**Conclusions:**

During the study period, prevalence was different among the two subtypes of RSV, and the average age of RSV-B-positive patients was higher than that of RSV-A. Co-infection rate tended to increase every year. RSVs cause mild as well as severe infections. There are reports of serious clinical progress as RSVs cause overlapping infections with other viruses and increase the risk of secondary bacterial infections. Thus, further research on RSV should be done.

## Background

Respiratory infectious diseases are a primary reason behind frequent inpatient and outpatient visits of infants and young children. Respiratory viruses cause lower respiratory tract infections or contribute to acute exacerbation of asthma [1]; the viruses can also cause severe respiratory infections in children with bronchial pulmonary dysplasia, immunodeficiency, or congenital heart disease. Major viruses responsible for respiratory infections are adenovirus, coronavirus, human rhinovirus, influenza virus, human metapneumovirus, parainfluenza virus, and respiratory syncytial virus (RSV) [2].

RSV is an RNA virus, which belongs to the genus *Orthopneumovirus*, and it is the most common cause of bronchiolitis and pneumonia in children under 1 year of age [3, 4]. Nearly all children are infected with RSV at least once before the age of two [5], and RSV bronchiolitis in infants is a major reason for their hospitalization [6]. Most people infected with RSV recover within one to 2 weeks, but it causes pneumonia in in older or immunocompromised individuals, which requires hospitalization. Individuals with congenital heart defects, premature infants, children immediately after open heart surgery, and immunocompromised patients (such as bone marrow transplant or organ transplant patients), also previously healthy children are severely affected by RSV infections [7, 8].

To date, there are no RSV vaccines or specific antiviral agents, and symptomatic treatment is under way. The most important treatment for acute bronchiolitis, mainly caused by the respiratory syncytial virus (RSV), is oxygen and hydration. Drug therapy is not recommended, and it improves clinical symptoms in the short term, but it does not affect cure [9]. Most adults have neutralizing antibodies for RSV, but reinfection is also common [10]. The epidemiological and clinical characteristics of RSV-A and B infections differ [11].

The clinical symptoms of most of respiratory viral infections are similar so we cannot easily distinct; thus, for early diagnosis of RSV infection it is important to identify the causative virus. Thus, a real-time PCR based method, which is faster than the conventional PCR method, is required for quick diagnosis and rapid treatment of RSV infections, which can also decrease antibiotic resistance.

In Korea, sample monitoring of patients with respiratory diseases has been conducted since 2011, and RSV infections have been reported to increase gradually [12]. In this study, RSV infections were diagnosed with a single-step real-time reverse transcription (RT)-PCR based method from samples monitored at a single institute between January 2012 and December 2018; these samples were analyzed according to the year and month of sample collection, and sex and age of the patients. The epidemic and co-infection patterns and the differences according to RSV subtypes were also analyzed. In addition, the relationship of RSV infections with temperature was analyzed using the Meteorological Agency’s climate data [13].

## Material and METHODS

### Respiratory specimens

We analyzed a total of 9010 respiratory specimens from all inpatients with respiratory symptoms (nasopharyngeal aspirate, nasal swab, throat swab), which were sent to the Department of laboratory medicine, Dankook university from January 2012 to December 2018 for identification of respiratory viruses via real-time RT-PCR. The samples were either immediately tested or refrigerated at 4 °C and tested within 24 h.

### Extraction of RNA

The collected specimens were treated with the QIAamp® MinElute® Virus Spin Kit (Qiagen, Hilden, Germany) for RNA extraction.

### Synthesis of cDNA (complementary DNA)

cDNA was synthesized using RevertAid™ First Strand cDNA Synthesis Kit (Fermentas, Ontario, Canada). The reverse transcription reaction was performed by mixing 50 ng of extracted RNA with random hexamers (0.2 μg/μl) at 25 °C for 5 min. RT buffer, 10 mM dNTP, RNase inhibitor (20 μg/μl), and reverse transcriptase (200 μg/μl) were added to the mixture (the final reaction volume was 20 μl), which was incubated at 37 °C for 90 min.

### Real-time RT-PCR

The synthesized cDNA was then amplified and probed for RVs with the AdvanSure™ RV real-time RT-PCR (LG Life Science, Seoul, Korea) according to the manufacturer’s instructions. Briefly, 5 μl of the cDNA was added to a tube containing 5 μl of primer probe mix and 10 μl of one-step RT-PCR premix. For the reverse transcription step, this mixture was incubated at 50 °C for 10 min. Denaturation was done at 95 °C for 30 s, followed by 10 cycles of PCR (15 s at 95 °C, 30 s at 53 °C, and 30 s at 60 °C). Subsequently, 30 additional cycles of PCR were completed for the detection of fluorescence signals (15 s at 95 °C, 30 s at 53 °C, 30 s at 60 °C). During the study, the same kits were used in the experiment and there was no change.

### Statistical analysis

The data used in this study are laboratory data of Cheonan Hospital of Dankook University. The hospital has an average of 900,000 inpatients and outpatients per year, and samples of suspected respiratory infections from January 1, 2012 to December 31, 2018. The results obtained by collecting and performing RT-PCR tests were used. All statistical analyses were performed using SPSS 26 program for windows. The characteristics of this study were frequency analysis and cross-analysis was used for positive patients and climatic factors were compared using the student t test.

RSV detected by real-time RT-PCR was analyzed based on co-infection, sex and age of the patients, and year and month of sample collection. A *p*-value of less than 0.05 was considered significant.

### Climate data

Climate data for the Cheonan region used in this study was provided by the weather data opening portal of the Korea Meteorological Administration.

## Results

In this study, we observed that the RSV detection rate was 12.8% (*n* = 1150/9010), the RSV-A detection rate was 7.1% (*n* = 639/9010), and the RSV-B detection rate was 5.8% (*n* = 521/9010). The median age of RSV-positive patients was 0.4 years (IQR 0.2–1.7), the median age of RSV-A-positive patients was 0.4 years (IQR 0.2–1.4), and the median age of RSV-B-positive patients was 0.6 years (IQR 0.2–1.9). RSV detection rate in males was 12.2% (*n* = 638/ 5242) and in females was 13.6% (*n* = 512/3768) (Table 1).

**Table 1:** Details of respiratory specimens during the study

Characteristics of specimens	Number of specimens	
Median age (IQR^a^)	2.7 (0.5–36.5)	
Patients	9010	
RSV positive patients (%)	1150 (12.8)	
Male specimens	5242	
RSV positive male patients (%)	638 (12.2)	
Female specimens	3768	
RSV positive female patients (%)	512 (13.6)	
	RSA-A	RSA-B
Median age (IQR^a^)	0.4 (0.2–1.4)	0.6 (0.2–1.9)
	Number (%)	Number (%)
Positive patients (%)	639 (7.1)	521 (5.8)
Positive male patients (%)	364 (6.9)	282 (5.4)
Positive female patients (%)	275 (7.3)	239 (6.3)

^a^*IQR* Interquartile range

Age = year

**Fig. 1:**
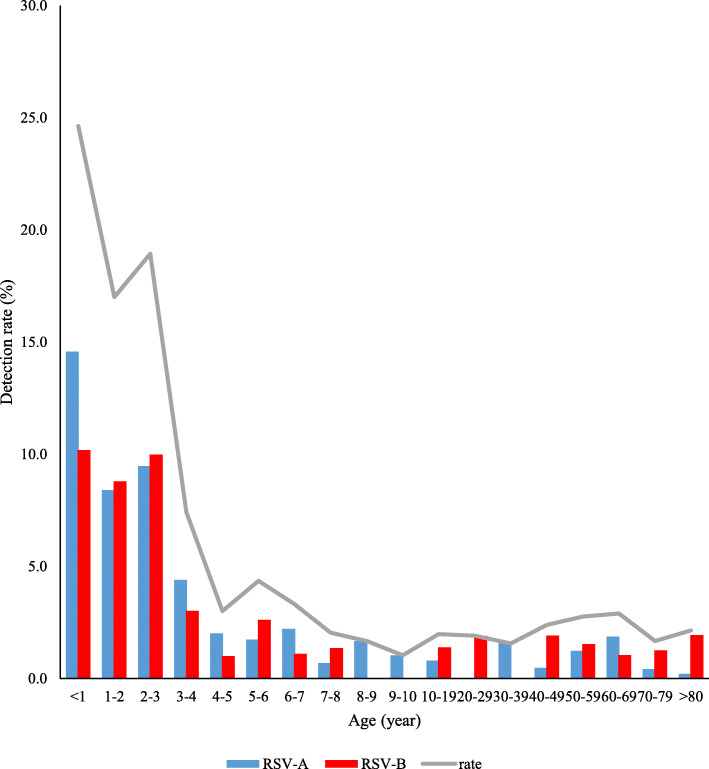
Detection rate of RSV in respiratory specimens aggregated by age

The detection rate in under 1-year-old patients was the highest at 24.6% (*n* = 740/3005), and the detection rate in under 9–10-years-old patients was the lowest at 1.0% (*n* = 1/97) (Fig. 1).

**Fig. 2:**
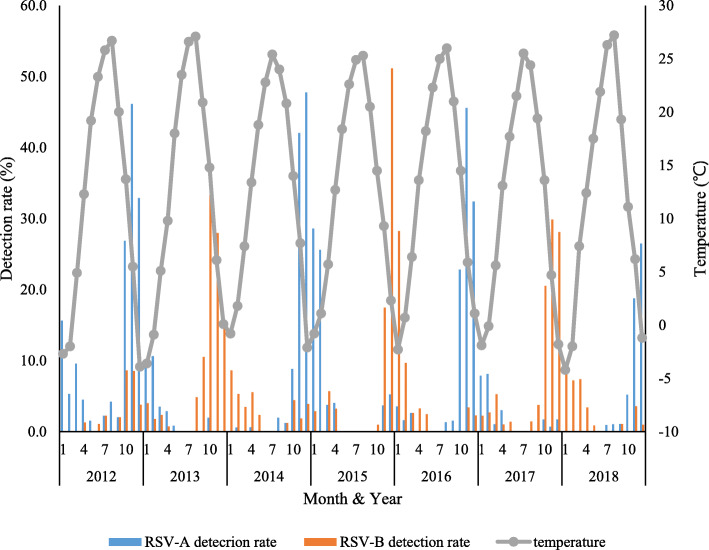
Detection rate of RSV in respiratory specimens aggregated by temperature

During the study period, infections caused by RSV-A and RSV-B dominated in alternate years; RSV-A infections dominated in 2012, 2014, 2016 and 2018 over RSV-B (Fig. 2).

**Fig. 3:**
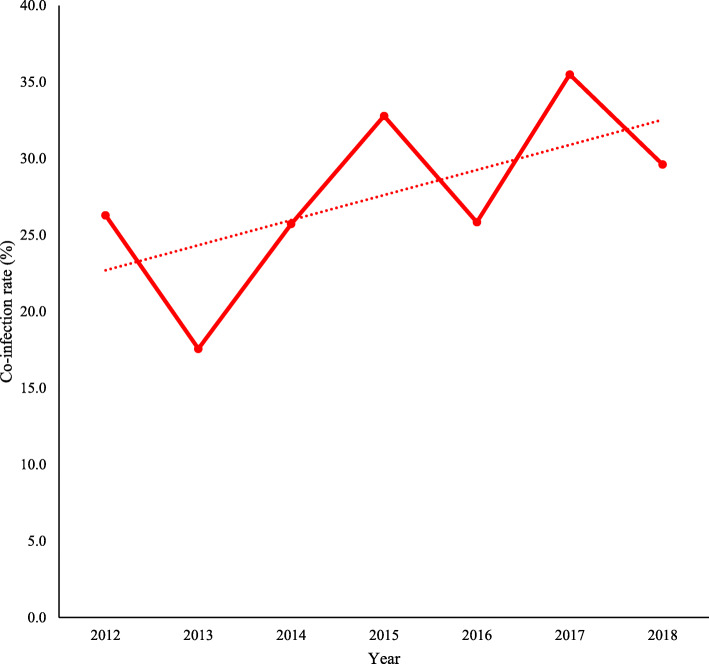
Annual co-infection rate trend for RSV from respiratory specimens

Co-infection rate was the highest in 2017 at 35.5% (*n* = 55/155) and the lowest in 2013 at 17.6% (*n* = 23/131) (Fig. 3).

**Fig. 4:**
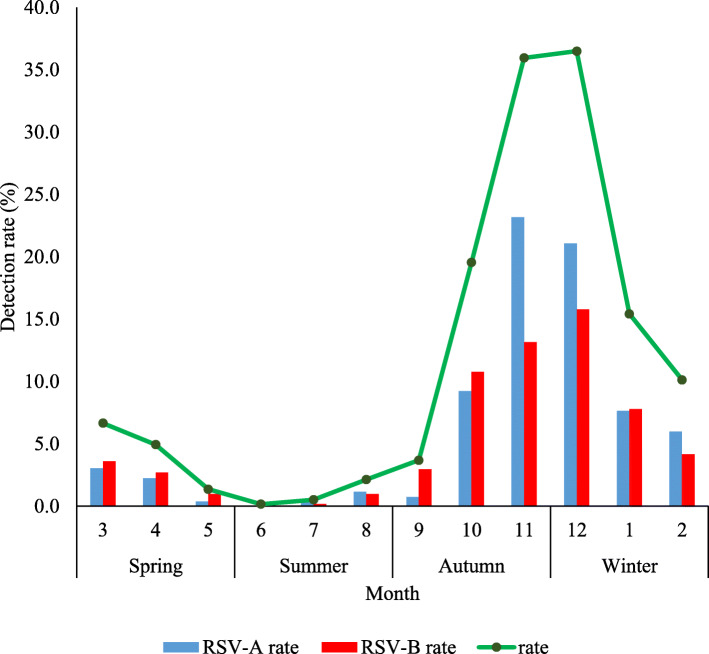
Detection rate of RSV in respiratory specimens aggregated by month and season

We observed that the detection rate of RSV-A and RSV-B together was the highest in December at 36.5% (*n* = 386/1058) and the lowest in June at 0.2% (*n* = 1/636). The detection rate of RSV-A was the highest in November at 23.2% (*n* = 192/829) and the lowest in June at 0% (*n* = 0/636). The detection rate of RSV-B was the highest in December at 15.8% (*n* = 167/1058), the lowest in June at 0.2% (*n* = 1/636) and July at 0.2% (*n* = 1/590) (Fig. 4). In addition, as a result of analyzing RSV-A, B and analysis of climatic factors, it is observed that the detection rate is high when the average temperature is low (Table 2).

**Table 2:** Differences in temperature and relative humidity by RSV-A, B detection (week)

Variable	RSV-A detection		RSV-A non-detection		*P*-value
	N	Mean ± SD	N	Mean ± SD	
Temperature (°C)	134	6.20 ± 8.32	231	15.96 ± 9.57	< 0.001
Relative Humidity (%)		66.44 ± 9.14		68.84 ± 9.68	0.019
Variable	RSV-B detection		RSV-B non-detection		*p*-value
	N	Mean ± SD	N	Mean ± SD	
Temperature (°C)	148	7.20 ± 8.43	217	15.91 ± 9.91	< 0.001
Relative Humidity (%)		67.23 ± 8.99		68.46 ± 9.90	0.217

The mean co-infection rate during the study was 27.7% (*n* = 319/1150). The co-infection rate of RSV-B was higher than that of RSV-A. Both RSV-A and RSV-B showed the most co-infection with *Rhinovirus* (Table 3). RSV-A had the highest double infection with *Rhinovirus* and *Coronavirus* OC43, and RSV-B had the highest double infection with *Adenovirus*. Of the 274 double infections of RSV identified, 138 were due to RSV-A and 143 were due to RSV-B. There were 7 co-infections of RSV-A and RSV-B. Of the 43 triple infections, 22 were due to RSV-A and 24 were due to RSV-B. Among the triple infections of RSV-A and RSV-B, 10 and 11 patients, respectively, were co-infected with *Adenovirus* and *Rhinovirus* (Table 4).

**Table 3:** Double infection of 12 species respiratory virus

	Influenzavirus-A	Influenzavirus-B	RSV-A	RSV-B	Metapneumo virus	Parainfluenza virus-1	Parainfluenza virus-2	Parainfluenza virus-3	Rhinovirus	Coronavirus 229E	Coronavirus OC43	Adenovirus
**Influenzavirus-A**												
**Influenzavirus-B**	10											
**RSV-A**	8	0										
**RSV-B**	7	2	7									
**Metapneumo virus**	5	5	6	6								
**Parainfluenza virus-1**	1	0	0	1	1							
**Parainfluenza virus-2**	1	0	1	4	0	0						
**Parainfluenza virus-3**	2	4	2	5	9	0	0					
**Rhinovirus**	21	11	**70**	**63**	46	31	11	78				
**Coronavirus 229E**	17	2	6	4	2	0	0	2	7			
**Coronavirus OC43**	10	4	**21**	12	4	1	0	2	19	2		
**Adenovirus**	16	8	17	**32**	22	5	4	25	228	5	13

**Table 4:** Triple Infection of RSV and respiratory virus

Pathogen			Specimen (No.)
**RSV-A**	Adenovirus	Coronavirus 229E	0
		Coronavirus OC43	3
		Influenzavirus-A	0
		Influenzavirus-B	0
		Metapneumo virus	0
		Parainfluenza virus-1	0
		Parainfluenza virus-2	0
		Parainfluenza virus-3	1
		Rhinovirus	10
		RSV-B	0
	Coronavirus 229E	Coronavirus OC43	0
		Influenzavirus-A	2
		Influenzavirus-B	0
		Metapneumo virus	0
		Parainfluenza virus-1	0
		Parainfluenza virus-2	0
		Parainfluenza virus-3	0
		Rhinovirus	0
		RSV-B	0
	Coronavirus OC43	Influenzavirus-A	0
		Influenzavirus-B	0
		Metapneumo virus	0
		Parainfluenza virus-1	0
		Parainfluenza virus-2	0
		Parainfluenza virus-3	0
		Rhinovirus	0
		RSV-B	0
	Influenzavirus-A	Influenzavirus-B	0
		Metapneumo virus	0
		Parainfluenza virus-1	0
		Parainfluenza virus-2	0
		Parainfluenza virus-3	0
		Rhinovirus	1
		RSV-B	0
	Influenzavirus-B	Metapneumo virus	0
		Parainfluenza virus-1	0
		Parainfluenza virus-2	0
		Parainfluenza virus-3	0
		Rhinovirus	0
		RSV-B	0
	Metapneumo virus	Parainfluenza virus-1	0
		Parainfluenza virus-2	0
		Parainfluenza virus-3	0
		Rhinovirus	1
		RSV-B	0
	Parainfluenza virus-1	Parainfluenza virus-2	0
		Parainfluenza virus-3	0
		Rhinovirus	0
		RSV-B	0
	Parainfluenza virus-2	Parainfluenza virus-3	0
		Rhinovirus	1
		RSV-B	0
	Parainfluenza virus-3	Rhinovirus	0
		RSV-B	0
	Rhinovirus	RSV-B	3
**RSV-B**	Adenovirus	Coronavirus 229E	1
		Coronavirus OC43	2
		Influenzavirus-A	0
		Influenzavirus-B	0
		Metapneumo virus	0
		Parainfluenza virus-1	0
		Parainfluenza virus-2	0
		Parainfluenza virus-3	0
		Rhinovirus	11
		RSV-A	0
	Coronavirus 229E	Coronavirus OC43	1
		Influenzavirus-A	2
		Influenzavirus-B	1
		Metapneumo virus	0
		Parainfluenza virus-1	0
		Parainfluenza virus-2	0
		Parainfluenza virus-3	0
		Rhinovirus	0
		RSV-A	0
	Coronavirus OC43	Influenzavirus-A	0
		Influenzavirus-B	0
		Metapneumo virus	0
		Parainfluenza virus-1	0
		Parainfluenza virus-2	0
		Parainfluenza virus-3	0
		Rhinovirus	2
		RSV-A	0
	Influenzavirus-A	Influenzavirus-B	0
		Metapneumo virus	0
		Parainfluenza virus-1	0
		Parainfluenza virus-2	0
		Parainfluenza virus-3	0
		Rhinovirus	1
		RSV-A	0
	Influenzavirus-B	Metapneumo virus	0
		Parainfluenza virus-1	0
		Parainfluenza virus-2	0
		Parainfluenza virus-3	0
		Rhinovirus	0
		RSV-A	0
	Metapneumo virus	Parainfluenza virus-1	0
		Parainfluenza virus-2	0
		Parainfluenza virus-3	0
		Rhinovirus	0
		RSV-A	0
	Parainfluenza virus-1	Parainfluenza virus-2	0
		Parainfluenza virus-3	0
		Rhinovirus	0
		RSV-A	0
	Parainfluenza virus-2	Parainfluenza virus-3	0
		Rhinovirus	0
		RSV-A	0
	Parainfluenza virus-3	Rhinovirus	0
		RSV-A	0
	Rhinovirus	RSV-A	3

## Discussion

During the study, the total RSV detection rate was 12.8% (*n* = 1150/9010) and the average age of patients was 4.1 years (median age: 0.4 years, range year). The average age of RSV-A infected patients was 2.9 years and the average age of RSV-B infected patients was 5.5 years.

We also observed an alternating pattern between RSV-A and RSV-B infections during the study period. In a study in Busan, Korea, RSV-A and B types showed an alternating trend similar to our results. Although host-immunity against RSV-B is active longer than that of RSV-A, RSV-A is considered to be more dominant [14], but more detailed studies, such as the study related to immunity in the population are needed.

In this study, we observed that the detection rate of RSV-B in January, March, April, May, June, and September was higher than that of RSV-A. Similar studies in Korea have reported that RSV-A prevailed between November and December, with RSV-B being dominant in January and February [13]. Studies conducted in temperate climates and RSV, such as in Korea, have reported to be prevalent between October and April [15–17]. Compared with similar studies in China over a similar period, the results do not show as clear alternating pattern [18].

The detection rate in the group of under 1-year-old patients was the highest at 24.6% (*n* = 740/3005). The average age of RSV-B infected patients was 5.5 years (median age: 0.6 years, range year), which was higher than the average age of RSV-A infected patients, 4.1 years (median age: 0.4 years, range year). Some studies have reported that RSV-B infections occur in patients older than the patients infected by RSV-A [19].

The co-infection rate of RSV-B was higher than that of RSV-A. We observed that except for the quadruple infections detected during the study period, the average age of the patients increased as the number of pathogens increased in co-infections. The highest rate of double infections was observed in the 80-year-old patients (80%, *n* = 8/10), followed by (50%, *n* = 1/2) the 20–29-year-old patients, and the (40%, *n* = 4/10) 10–19-year-old patients. The results are similar to other studies that co-infection is most common in RSV, rhinovirus and bacterial pathogens [20].

Co-infection rate tended to increase gradually. The co-infection rate in 2017 was the highest at 35.5% (*n* = 55/155) and the lowest in 2013 was 17.6% (*n* = 23/131). Follow-up studies of duplicate infections with respiratory bacteria as well as duplicate infections between respiratory viruses are also needed.

Previous studies have reported respiratory infections in up to 11.5–45.4% of the children, with the most common causal organisms being RSV and rhinoviruses [21, 22]. This differs depending on several factors, such as age, detection time, detection method, and detection area. Co-infection with RSV and *Metapneumovirus* has been reported to increase the relative risk of pediatric intensive care unit (ICU) admission of patients by 10-fold [23]. It has been reported that co-infection with RSV and *Rhinovirus* shows a five-fold increase in the severity of RSV infections [24].

The prevalence of RSV is generally reported to be high in places with low mean temperature and high relative humidity. Some subtypes of RSV are prevalent at equal rates, but one subtype prevailed [13], and some studies reported that RSV-B infections occurred in patients older than the patients that were infected with RSV-A [19]; however, RSV-A has been reported to have higher severity than RSV-B infections [25, 26].

In addition to being recognized as a primary cause of mild as well as severe respiratory infections, reports show serious clinical implications of these infections, as RSVs cause overlapping infections with other viruses and increase the risk of secondary bacterial infections in patients with low immunity. Thus, research on RSVs should be continued by broadening the parameters under consideration.

This study is a retrospective study that has not been conducted according to the clinical symptoms of the test subjects. We could not analyze the genotypes of the viruses and their distribution. Analyzing the prevalent RSV genotypes in each community is likely to improve the ecological understanding of RSV infections. Further research is required to address these issues.

## Abbreviations

real-time RT-pcr: real-time reverse transcription-pcr
RSV: Respiratory syncytial virus
cDNA: complementary DNA; intensive care unit, ICU
